# Optimization of UHF RFID Five-Slotted Patch Tag Design Using PSO Algorithm for Biomedical Sensing Systems

**DOI:** 10.3390/ijerph17228593

**Published:** 2020-11-20

**Authors:** Ibtissame Bouhassoune, Abdellah Chehri, Rachid Saadane, Khalid Minaoui

**Affiliations:** 1 LRIT Laboratory, Mohammed V University, Rabat 10106, Morocco; khalid.minaoui@um5.ac.ma; 2 Department of Applied Sciences, University of Quebec in Chicoutimi, Chicoutimi, QC G7H 2B1, Canada; 3 SIRC/LaGeS-EHTP, EHTP Km 7 Route, El Jadida 20230, Morocco; saadane@ehtp.ac.ma

**Keywords:** radio frequency identification (RFID), UHF band, wearable RFID tag, PSO algorithm, tag robustness

## Abstract

In this paper, a new flexible wearable radio frequency identification (RFID) five-shaped slot patch tag placed on the human arm is designed for ultra-high frequency (UHF) healthcare sensing applications. The compact proposed tag consists of a patch structure provided with five shaped slot radiators and a flexible substrate, which minimize the human body’s impact on the antenna radiation performance. We have optimized our designed tag using the particle swarm optimization (PSO) method with curve fitting within MATLAB to minimize antenna parameters to achieve a good return loss and an attractive radiation performance in the operating band. The PSO-optimized tag’s performance has been examined over the specific placement in some parts of the human body, such as wrist and chest, to evaluate the tag response and enable our tag antenna conception in wearable biomedical sensing applications. Finally, we have tested the robustness of this tag by evaluating its sensitivity as a function of the antenna radiator placement over the ground plane or by shaping the ground plane substrate for the tag’s position from the human body. Our numerical results show an optimal tag size with good matching features and promising read ranges near the human body.

## 1. Introduction

Recently, significant innovations in radio frequency identification (RFID) biomedical sensing engineering have allowed the emergence of a new generation of flexible and miniaturized electronic devices. These devices can be placed or implanted into the human body for healthcare applications [1,2].

The RFID sensing system consists of simple sensors embedded into RFID transponders and a specific reader able to monitor and track remote objects. Various active, semi-passive, and passive tags have added sensors into their design, allowing them to process information and transmit it to readers [1]. The interface between the wireless sensor tag and the reader is the antenna, which represents an important part of wireless communication and should be designed carefully to suit healthcare sensing applications [3].

The most recent RFID sensing works were focused on the characterization and design of wearable RFID sensors devices used as a wristband for patients. In these works, different techniques and tests have been considered for several types of antenna. Anatomical models of the human body, tissue-equivalent liquids, and animals have been used to analyze and evaluate human body tissues (skin, fat, muscle, and bones) on tag antenna radiation. Indeed, human body tissues’ electromagnetic properties affect the propagation, reflection, attenuation, and other behaviors of electromagnetic fields around the body. These properties depend strongly on the types of tissue and frequency. Furthermore, if the wearable RFID tag is nearby the body (on-body), the radiation performances and matching features (e.g., gain and matching impedance) of the tag antenna will be reduced [4,5].

The researches have widely addressed the problem of human tissue effects on the on-body antennas radiation efficiency. The goal is to decouple the antenna radiation part from the human body by using a thick ground plane to electrically isolate the antenna from the body [6,7], or by applying the open slot cavity concept in the patch antennas design [8]. Additionally, the antennas with only one layer of non-conducting substrate (with high permittivity) have been used to withstand the body effects, such as the dipole [9], the dual loop [10], and the meandered double loop tag [11]. Although these conceived tags present low profile, compact and flexible structures, they are influenced by many factors such as the location where the RFID tag is placed, the bending form of vital tissues, the movement of the human body, and its distance to the tag position.

The human body proximity significantly modifies on-body tags’ performance in real-world applications. The antenna–body distance changes randomly due to the natural wearer movements [12]. The robustness of wearable ultra-high frequency (UHF)-band planar inverted-F antennas (PIFAs) for the body–antenna separation and human tissue dispersion is reported in [13]. The performance of their antennas was addressed through numerical investigation. The authors also provided a criterion for the selection of the ground plane shape. They concluded that ground plane enlarging is more effective at the antenna border sections where the electric energy density exhibits a peak [14].

Other analytical approaches have been introduced to optimize the antenna’s radiation performances and matching features, mainly based on nature-inspired metaheuristic optimization algorithms. In particular, the genetic algorithm (GA), the particle swarm optimization (PSO) algorithm, and the ant colony optimization (ACO) are mostly used to deal with the antenna optimization design challenges [15,16]. The PSO optimization is one of the powerful algorithms used today to optimize the antenna performances, such as reducing the antenna size, achieving good matching features, and enhancing bandwidth [17,18]. An increase of the bandwidth and a reduction of the conventional antenna size have been obtained using a modified PSO algorithm and MATLAB and IE3D simulator [19].

In this paper, the Particle Swarm Optimization (PSO) with a curve fitting has been used to optimize the proposed UHF RFID patch with a five-shaped slot tag antenna placed on a human arm phantom. Our conceived tag has a low profile, compact structure, and flexible bio-silicone substrate with high permittivity. The multiple slots of the proposed antenna permit the integration of sensors and other electronic components. In our simulations, we have considered the anatomical structure of various parts of the human body to assess our proposed tag’s adaptability in different human body regions. Additionally, we have discussed our conceived tag’s performance to show a reasonable compromise between the robustness of our tag and its overall dimension.

The paper is organized as follows. In Section 2, we introduce the geometrical structure of the conventional proposed RFID tag antenna attached to the human arm model; we give a brief description of the PSO concept and the different steps used for the optimization. Additionally, we present our results obtained by of PSO code with curve fitting applied to the proposed tag antenna. The robustness of the PSO-optimized tag and effects of the cylindrical model of human arm phantom to the antenna characteristics are evaluated and presented in Section 3. Section 4 investigates the conceived antenna performances in other regions of the human body and presents the calculated tag reading distance from different human body parts. Finally, the concluding notes are given in Section 5.

## 2. Optimization of RFID Five-Slotted Patch Tag by PSO

To establish reliable and efficient RFID tags for biomedical applications, the wearable device, e.g., the medical device needs to provide accurate data transmission and low latency. Several frequency bands have been assigned to medical data transmission. Different countries have different criteria and certifications governed by regulatory agencies to use medical data transmission. In this work, 915 MHz was chosen to cover all potential medical frequency bands.

One major challenge to design RFID tags for biomedical applications is the miniaturization of long-lasting devices, which requires low profile antennas that can be easily worn on the human body.

This section presents the novel design of the patch antenna with five-slot attached to the human arm. In the first case, the proposed RFID tag antenna’s geometrical parameters have been performed by manual optimization using HFSS solver (conventional RFID tag antenna).

We have optimized this traditional tag using particle swarm optimization by selecting the antenna’s parameters as a variable. Besides, the selected parameters have been optimized using curve fitting and MATLAB code. All simulations performed in this work are via the electromagnetic simulators HFSS and CST software (Ansys, Inc. Canonsburg, Pennsylvania, PA, USA) [20,21].

### 2.1. On-Body Antenna Design

The conceived tag includes a patch radiator with five shaped slots. The choice impacts the antenna impedance and permits to achieve an inductive reactance. It also protects the antenna from the radiation losses caused by human skin. It includes an NXP UCODE G2XM microchip with complex impedance (Z = 34-j 142 Ω) [22]. Moreover, the proposed sensor tag will be placed around the human arm’s layered anatomical phantom at 915 MHz. This model consists of stratified parallelepiped boxes with defined properties such as thickness, dielectric constant, and conductivity.

**Figure 1:**
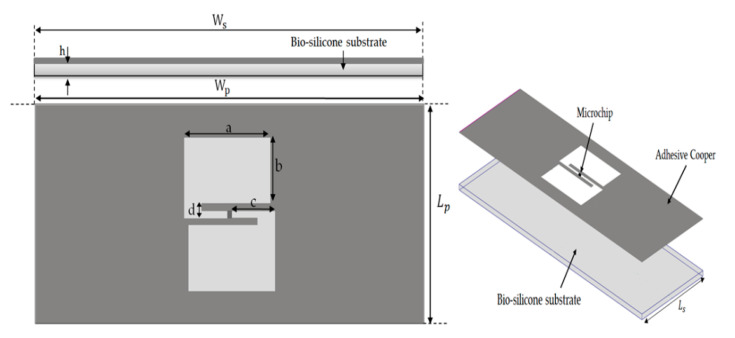
Geometrical design of conventional radio frequency identification (RFID) five-slotted patch tag.

The layout and geometrical dimensions of the proposed patch with a five-shaped slot tag are respectively given in Figure 1 and Table 1. The tag antenna is designed by using 0.035 mm of adhesive copper and 1 mm of the bio-silicone substrate with relative permittivity of 2.5 and conductivity of 0.005 S/m at 915 MHz. The antenna geometrical parameters are given in Table 1.

**Figure 2:**
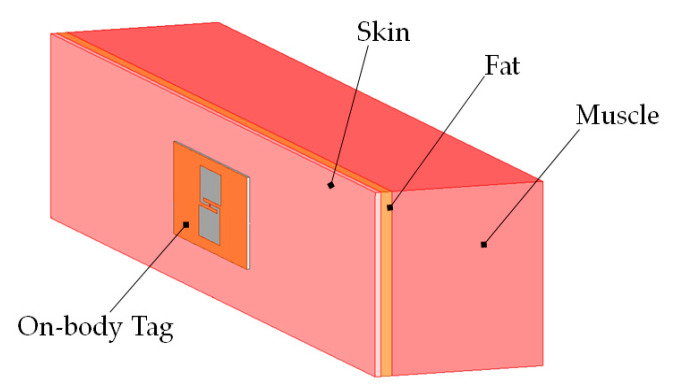
Simple human arm phantom model (310 mm × 60 mm × 60 mm).

The electrical properties of the human arm (skin, fat, muscle) phantom at 915 MHz are shown in Table 2 [23]. A numerical phantom has been added to the simulation scenario to analyze the body–antenna coupling. We have chosen a three-layer model, composed of a skin layer (2-mm thick), a fat layer (4-mm thick), and a muscle layer (54-mm thick) (see Figure 2) [24].

### 2.2. Simulations Results of a Conventional Tag and Discussion

This subsection presents the simulated results of the reflection coefficient, antenna input impedance, the gain, and the radiation pattern of the initially desired geometry of five slotted patch tag antennas. We then analyze the matching features of this conventional antenna to examine its performance near the human arm.

**Figure 3:**
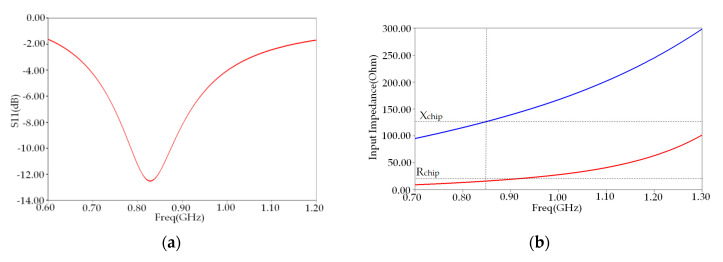
(**a**) Reflection coefficient S11 and (**b**) antenna input impedance versus frequency of conventional tag.

Figure 3a shows the reflection coefficient S11 of our conventional tag placed on the planar model of human arm phantom versus frequency in the UHF band. We notice that the maximum reflection coefficient S11 has a value of −12.49 dB at the resonance frequency of 865 MHz. The maximum bandwidth (S11 < −10 dB) of the proposed tag antenna was 12.48% (780–880 MHz), which does not cover the whole UHF band.

Figure 3b shows the comparison of input reactance and resistance between the conventional antenna and microchip. We note that the antenna’s input resistance in the resonant frequency 865 MHz differs from microchip resistance, while its corresponding reactance remains unchanged.

**Figure 4:**
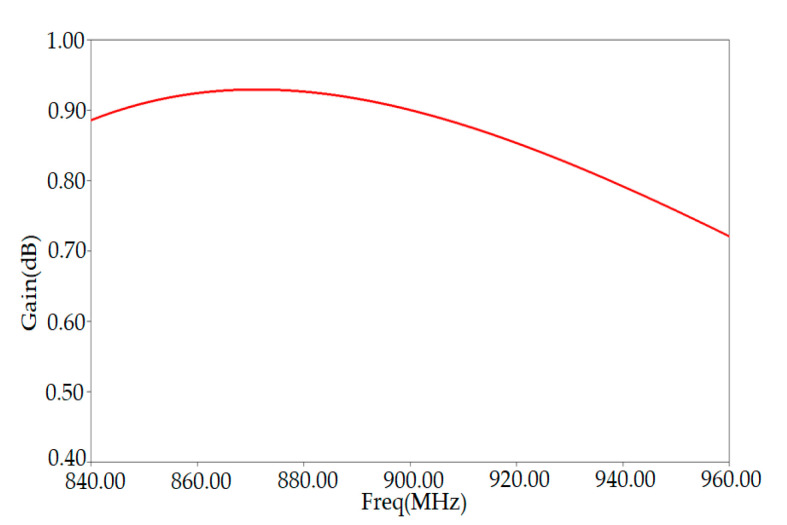
Gain versus frequency of RFID conventional tag antenna placed on human arm.

Figure 4 shows the simulation results of the tag antenna gain as a function of the frequency. We can extract the maximum value of the gain from the plot, which is 0.92 dB obtained around the resonance frequency of 865 MHz. Our proposed tag’s radiation performance (peak gain) is good in the proximity of the human arm phantom.

**Figure 5:**
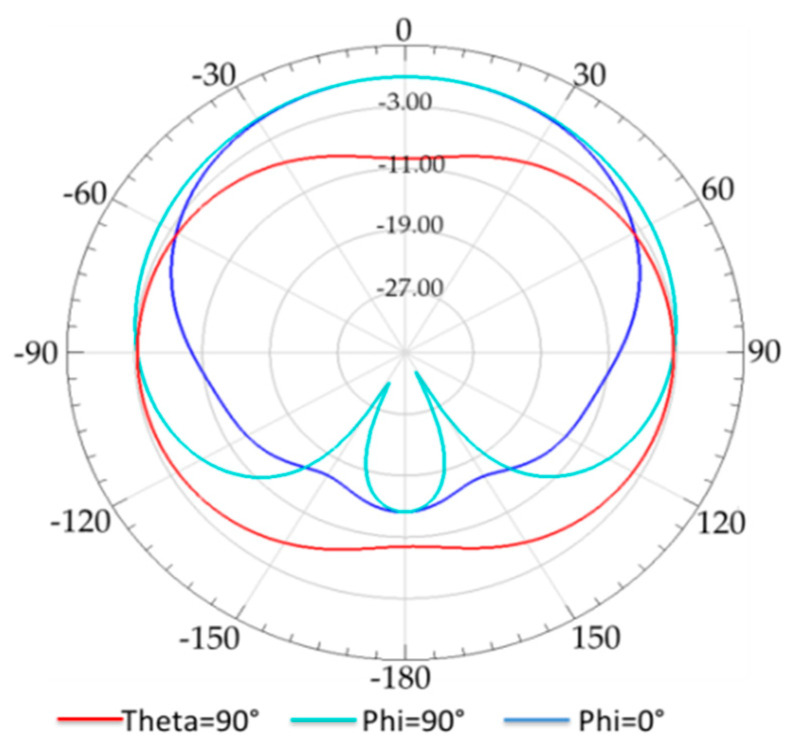
Radiation pattern of conventional RFID tag placed on human arm phantom at 865 MHz.

The 2D simulated peak gain radiation pattern of the conventional tag antenna at 865 MHz is depicted in Figure 5, and it shows that the radiation of the antenna is nearly omnidirectional in xz (phi=0°), yz (phi=90°), and xy(theta=90°). It is clear from this figure that the human arm tissues absorb some of the received electromagnetic waves.

### 2.3. Optimization by PSO

#### 2.3.1. PSO Algorithm

Particle swarm optimization (PSO) is a nature-inspired metaheuristic optimization algorithm developed in 1995 by J. Kennedy and R. Eberhart [25].

The PSO concept shares many similarities with evolutionary computation techniques such as Genetic Algorithms (GA) and Ant Colony. These algorithms are inspired by the social behavior of a swarm of bees or birds while searching for food [26]. The PSO is used to solve various optimization problems in several research fields, particularly antenna and electromagnetism [27]. It is based on individuals named particles. Each particle, which belongs to a swarm, flies through the N-dimensional problem space by following its personal flying experience and the other particles. Each particle tries to locate its position by utilizing the current location, current velocity, the distance between the current position and the personal best location ($p_{best}$), the distance between the current position and the global best location ($g_{best}$). The position of the best objective function (fitness) value personally discovered by a particle is called $p_{best}$ (personal best), and the position of the best fitness function found by the swarm is called $g_{best}$ (global best) [25].

Each particle in the population is associated with an adaptable velocity according to which it moves in the space. Further, the particle experiences are accelerated by two factors $C_{1}$ and $C_{2}$, and two random numbers generated between [0, 1], while the present movement is multiplied by an inertia factor w varying between [$w_{min}$; $w_{max}$].

Initially the population of size N and dimension D is generated randomly, each particle is given as $X_{i}\left(t\right)=\left[X_{1}\left(t\right);X_{2}\left(t\right);\ldots \ldots ;X_{i}\left(t\right);D\right]$, and also each particle is associated with a velocity vector denoted as $V_{i}\left(t\right)=\left[V_{1}\left(t\right);V_{2}\left(t\right);\ldots \ldots V_{i}\left(t\right)\right]$.

The velocity expression of the particle is described as follows [25,27].

(1) $$V_{i}\left(t+1\right)=W\times V_{i}\left(t\right)+C_{1}\times \delta_{1}\times \left(X_{P_{best}}\left(t\right)-X_{i}\left(t\right)\right)+C_{2}\times \delta_{2}\times \left(X_{g_{best}}\left(t\right)-X_{i}\left(t\right)\right)$$

where $V_{i}\left(t\right)$ is the velocity of the particle in the ith iteration; $X_{i}\left(t\right)$ the position of particle in the ith iteration; *t* is the current iteration; *W* is the weight constant that reduces linearly from 0.9 to 0.6; $C_{1}$ and $C_{2}$ are arbitrary constants generally set at 2.0; $\delta_{1}$ and $\delta_{2}$ are arbitrary functions distributed between 0 and 1.

After each iteration, the current position of the particle is generated by the following equation [27]:

(2) $$X_{i}\left(t+1\right)=X_{i}\left(t\right)+V_{i}\left(t+1\right)$$

The weighting function utilized in Equation (7) is given as follows.

(3) $$w=w_{Max}-\frac{\left(w_{Max}-w_{Min}\right)\times Iter}{Max_{Iter}}$$

where $w_{Max}$ is the initial weight; $w_{Min}$ is the final weight; $Max_{Iter}$ is the maximal iteration count; and Iter is the current iteration count.

#### 2.3.2. PSO Optimization Procedure

The selected antenna parameters to be optimized, such as length, width, and dimensions of five slot-shaped patch, represent the particle’s position, and a set of such positions should be taken initially. Then, the fitness of each position should be evaluated based on an objective function, which represents a function of position, and other antenna parameters should be considered as input parameters of PSO code. The objective function of optimization is to reach a good matching between the antenna and microchip by minimizing the reflection coefficient of our conventional antenna by changing seven key antenna parameters ($L_{p},\;W_{p}$, parameters of slot (a and b), c, d, and thickness h).

The fitness function represents the objective function to be examined, is obtained by calculating the simulated results, and is transferred to MATLAB in the form of data for evaluation. The optimization is executed using 100 swarm size for 1000 iterations. The fitness function represents the objective function to be examined and is defined as follows.

(4) $$Fitness\;function=\;\{\begin{array}{l} 1,\;if\;S_{11}\left(f_{i}\right)\leq -10\;\mathrm{dB}\\ 0,\;if\;S_{11}\left(f_{i}\right)\geq -10\;\mathrm{dB} \end{array}$$

with $f_{i}$ is the resonant frequency of the designed antenna.

The minimized reflection coefficient (fitness function) should be less than −10 dB. The HFSS solver and MATLAB are applied consistently. Visual Basic Script (VBS) links these two software by transferring the simulated reflection coefficient data from HFSS to MATLAB. The curve fitting is used to form a relationship between the variable parameters and the reflection coefficient (fitness function).

**Figure 6:**
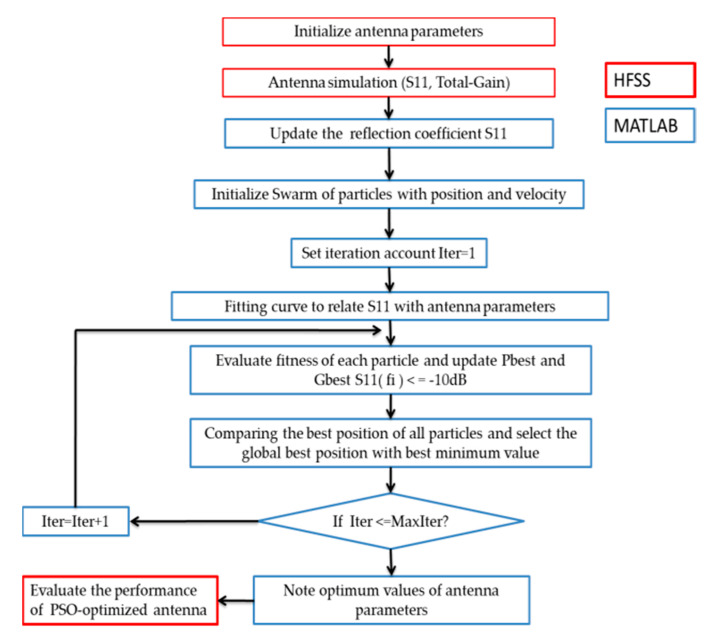
Flow chart of our antenna optimized by particle swarm optimization (PSO) method. HFSS: High Frequency Structure Simulator Software. MATLAB: matrix laboratory, proprietary multi-paradigm programming language and numerical computing environment developed by MathWorks.

A detailed flowchart of the PSO technique for the antenna parameters optimization is shown in Figure 6.

#### 2.3.3. Curve Fitting

We have used MATLAB Toolbox (curve fitting) to find the polynomial relationship between all varying parameters and the reflection coefficient S11. Each antenna parameters (Lp, Wp, Ls, Ws, a, b, d, c, h) are changed within its defined range while other Table 3 are kept constant. We have varied the patch and ground plane’s length and width in the same way to follow the structure of the initially desired geometry of the five-slot patch antenna. The simulation for different shapes of our designed antenna has been performed. The calculated reflection coefficient data is transferred to MATLAB. Using curve fitting, each reflection coefficient parameter can be described as a polynomial function transferred back to the PSO code, which then provides us the optimized fitness functions for each antenna.

The following polynomials Equations (5)–(10) obtained from curve fitting are given as follows.

(5) $$S11\left(L_{p}\right)=0.0002727L^{3}-0.06231L^{2}+\;4.579L-117.5$$

(6) $$S11\left(W_{p}\right)=-\;0.0009117w^{3}+0.273{\;w}^{2}-26.49w+811$$

(7) $$S11\left(a\right)=-0.02635{\;a}^{4}+1.265a^{3}-17.66a^{2}+91.04\;a-158$$

(8) $$S11\left(b\right)=0.0001023b^{4}-0.01348b^{3}+0.642b^{2}-12.92b+73.97\;$$

(9) $$S11\left(c\right)=-0.1653b^{4}+4.974b^{3}-48.9b^{2}+188.6b-260.7$$

(10) $$S11\left(d\right)=-1.1785b^{4}+14.9835b^{3}-58.897b^{2}-28.675b+10.56\;$$

(11) $$S11\left(h\right)=-2.314b^{4}+16.51b^{3}-24.75b^{2}-23.2b+5.501$$

#### 2.3.4. Optimized Results and Discussion

We have used the equations above (5)–(11) and the fitness function. The PSO code has been executed in MATLAB. We obtained the optimized values of variable parameters Lp, Wp, Ls, Ws, a, b, d, c, h after completing 1000 iterations with 100 particles. The comparison between the initial value and optimized value of parameters is presented in Table 4. The antenna with optimized value has been simulated in HFSS solves. The comparative results are shown in Table 5.

**Figure 7:**
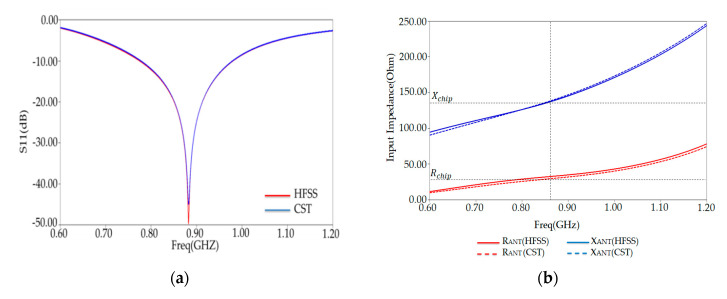
(**a**) Reflection coefficient S11 and (**b**) antenna input impedance versus frequency of PSO-optimized placed on human arm in HFSS and CST solvers.

Figure 7a shows the reflection coefficient, which describes how well the antenna is conjugate matched to the chip. The maximum bandwidth of our optimized tag simulated by HFSS and CST solver has been obtained 23.80% (770–980 MHz) at 882 MHz with a reflection coefficient −50 dB. Our PSO-optimized tag antenna can be functional within the universal UHF RFID band.

To validate the above results, in Figure 7b, we present the input impedance of PSO-optimized antenna placed on human arm phantom—both the input reactance and resistance of the antenna match well with those of the tag chip.

**Figure 8:**
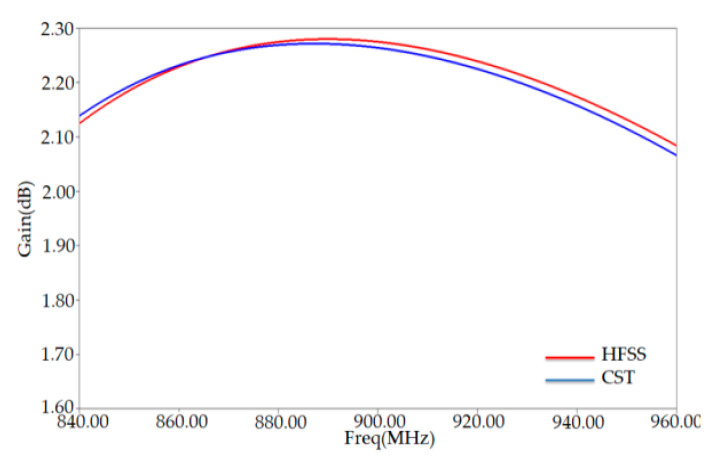
Gain versus frequency of PSO-optimized placed on human arm in HFSS and CST solvers.

The maximum gain extracted from Figure 8 has a value of 2.27 dB at around 882 MHz. This value is higher than that of a conventional tag (0.92 dB) obtained at the frequency 865 MHz. At this frequency (865 MHz) of our gain is around 0.92 dB.

To check HFSS simulation results of our PSO-optimized antenna characteristics, such as the S11, antenna input impedance, and the gain, we have compared another simulator (CST simulator). This comparison is shown in Figure 7 and Figure 8, indicating nearly similar matching features and radiation performance. The slight differences between the results of the two simulators can be attributed to the difference between the numerical codes of each one.

## 3. Robustness Analysis of PSO-Optimized Tag

### 3.1. The Effects of Different Ground Plane Materials

**Figure 9:**
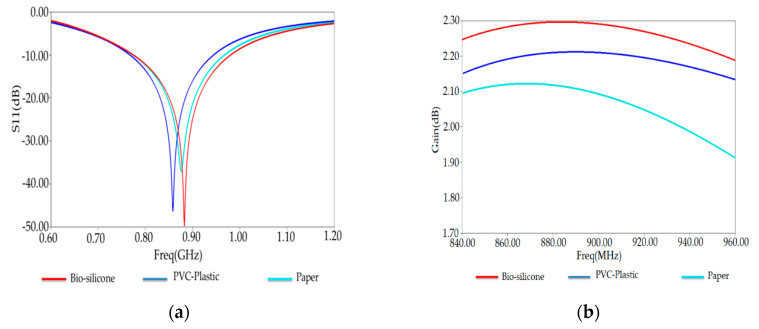
(**a**) Reflection coefficientS11 and (**b**) Gain of optimized tag antenna for different substrates.

Effects of materials on impedance matching and radiation performances of the proposed antenna have been investigated. Figure 9a,b shows respectively the reflection coefficient of the antenna for different substrates materials, Bio-silicone, PVC-plastic (ε = 2.7, loss = 0.007), and paper (ε = 3.2, loss tangent = 0.05) at 915 MHz. Remarkably, the conceived antenna with bio-silicone substrate presents good radiation performances and well-matching features.

### 3.2. The Effect of Bending PSO-Optimized Tag

**Figure 10:**
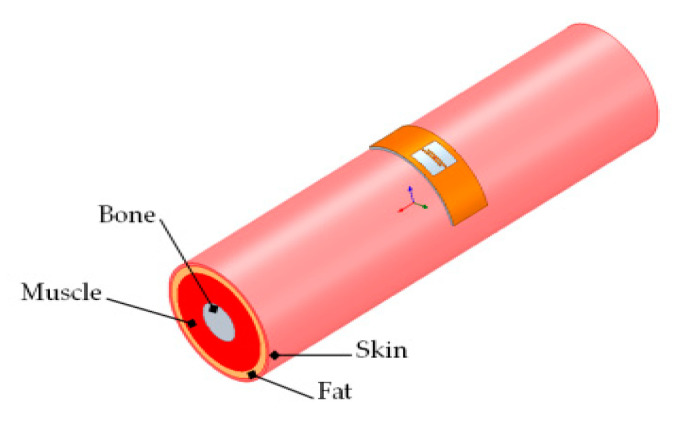
Human arm cylindrical model [28].

The PSO-optimized tag antenna is simulated through a human arm model with a four-cylindrical layer (skin, fat, muscle, and bone) approximating those of the human body, as shown in Figure 10. The electrical properties of tissues are taken from the database in ref [22] at 915 MHz. The dimension of the cylindrical layer model given in [28] has been considered in the simulated environment are listed in Table 6.

**Figure 11:**
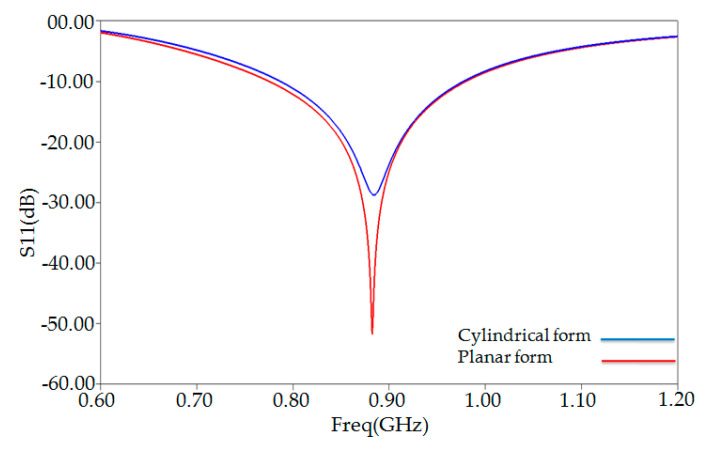
Reflection coefficient S11 (dB) versus frequency of RFID PSO-optimized tag on the layered cylindrical model and planar model of human arm phantom.

**Figure 12:**
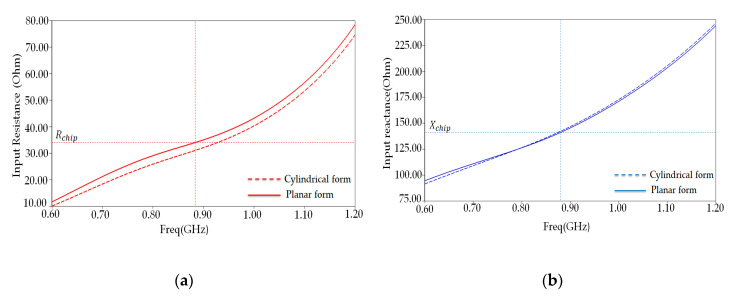
PSO-optimized antenna input impedance (Ohm) in a cylindrical form and planar form: (**a**) resistance and (**b**) reactance.

This model helps us to see what happens to the antenna characteristics compared to the planar model. Figure 11 shows the proposed antenna’s simulated reflection coefficient when the antenna is simply in planar form and when it is bent to conform to a cylindrical human arm model. As depicted in this figure, bending the proposed tag antenna will only slightly affect the impedance matching, clearly explained in Figure 12a,b, showing a deviated somewhat input resistance value around 882 MHz, whereas its corresponding reactance remains unchanged. As shown in Figure 12, the proposed antenna value (37 ohms) input resistance around the resonance frequency is slightly deviated, whereas its corresponding reactance (142 ohms) stays unchanged.

**Figure 13:**
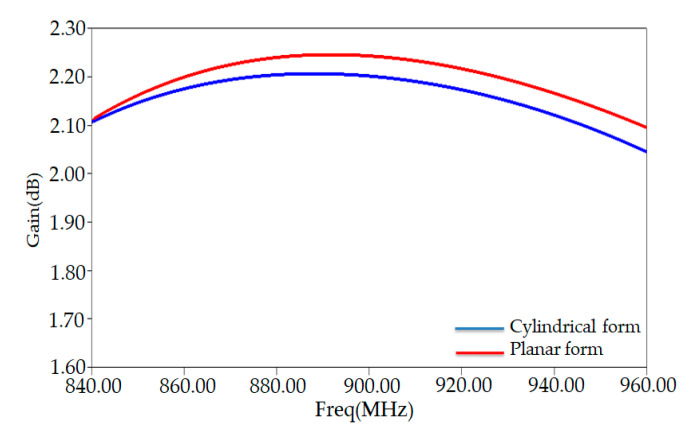
Comparison of gain of proposed tag between the cylindrical and planar model of human arm.

Figure 13 shows the comparison of gain between the antenna in planar and bending form. As depicted in Figure 13, the obtained gain is not affected by bending the tag antenna structure—nearly similar results obtained from these two models.

### 3.3. PSO-Antenna Robustness to Human-Body Proximity

**Figure 14:**
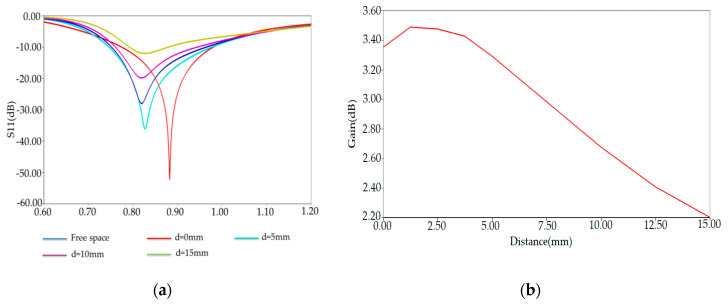
(**a**) Simulated antenna reflection coefficient S11 and (**b**) Realized-Gain for a various distances from human arm phantom at 882 MHz.

The reflection coefficient S11 and the gain of the designed PSO-optimized tag antenna in free space and proximity from the human arm phantom model are shown respectively in Figure 14a,b.

The plots of S11 and gain demonstrate a less detuning of an overall proposed antenna performance when the human body is placed nearby of human arm model. This figure shows the comparison between free space and the human body of the measured antenna reflection coefficients S11 for different antennas. It is seen that the resonant frequencies of antennas shift only slightly, implying the detuning effects of the human body are negligible in this scenario.

### 3.4. PSO-Optimized Antenna with Extended Ground Plane Robustness to Human-Body Proximity

To improve the antenna robustness for the distance from the human body, the PSO-optimized antenna’s robustness has been tested in terms of electric density distribution to evaluate the optimal ground plane extension.

**Figure 15:**
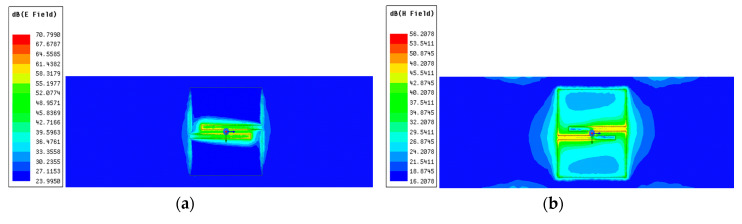
(**a**) Front view of electric field distribution and (**b**) magnetic field distribution of a PSO-optimized antenna radiating at 882 MHz in a tissue emulating arm phantom.

The electric and magnetic field distribution of the antenna, simulated in the bio-silicone dielectric ground plane, are shown respectively in Figure 15a,b for the case when the antenna is placed directly on the human body and at the resonance frequency of the antenna (882 MHz).

As depicted in Figure 15a,b, the electric field presents a peak in the antenna’s center. In contrast, the magnetic field distribution shows the peak in the antenna center, along the two feeding lines and close to the antenna border toward the *y*-axis (Figure 15b). According to the reference [29], to choose the direction of optimal ground plane extension, the ground plane’s enlargement must follow the region of the high distribution of the electric field.

The PSO-optimized antenna’s ground plane has been enlarged toward the region of electric energy density by considering the extended dimension ΔL1 = ΔL2 = 25 mm and ΔL3 = 12.5 mm according to the approach presented in [15].

**Figure 16:**
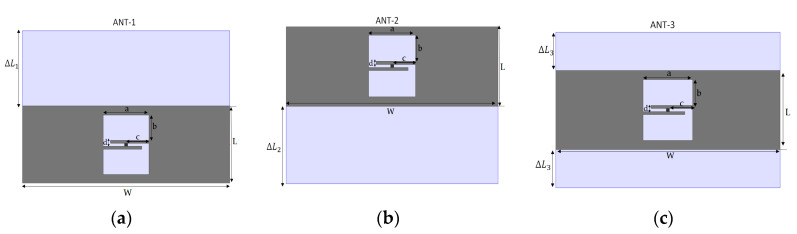
The PSO-optimized tag with an extended ground plane. (**a**) the antenna with an extended ground plane toward the up region close to an electric energy density peak. (**b**) the antenna with an extended ground plane toward the down region close to an electric energy density peak. (**c**) the antenna with an extended ground plane toward the middle region close to an electric energy density peak.

Figure 16 presents the three proposed antenna versions that have been considered for investigation. The layout (both the silicone substrate and the metallization) has been extended toward the regions close to an electric energy density:

− ANT-REF (Figure 1) represents the PSO-optimized antenna with an extended configuration (both ground plane and metallic layer have been extended) toward the regions close to an electric energy density.
− ANT1 (Figure 16a) represents the antenna with an extended ground plane toward the up region close to an electric energy density peak.
− ANT2 (Figure 16b) represents the antenna with an extended ground plane toward the down region close to an electric energy density peak.
− ANT3 (Figure 16c) represents the antenna with an extended ground plane toward the middle region close to an electric energy density peak.

**Figure 17:**
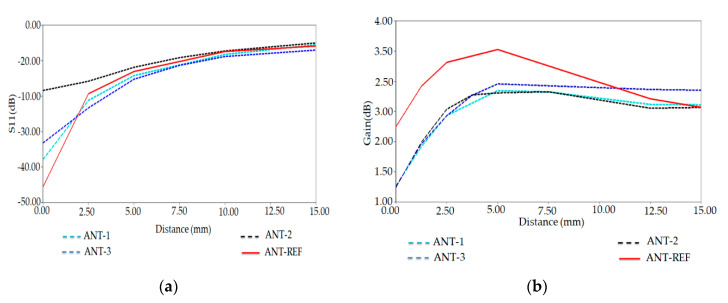
(**a**) Reflection coefficient S11 and (**b**) Gain versus distance from human arm for three antenna versions at 882 MHz.

From the simulation results presented in Figure 17a,b, we see that the ANT-REF profile is better and then the ANT-1, ANT-2, and ANT-3 in terms of reflection coefficient and gain. The reflection coefficient and realized-gain of Ant-REF versus the distance from the human arm phantom reached the value of −50 dB and 3.5 dB, respectively.

Whereas the ANT-1, ANT-2, and ANT-3 present good radiation performance and matching features when varying the antenna-body distance, their properties remain less than those of ANT-REF. This example demonstrates that an extension of the complete configuration of the antenna (patch and ground plane) toward the regions close to the peaks of the electric energy density helps improve the robustness of the antenna performance for the variation of the antenna-body distance.

## 4. Comparison of PSO-Optimized Tag Performance for Different Human Body Models

Our numerical simulations were performed for PSO-optimized five slotted patch tags in other regions of the human body to validate our design.

From design perspectives, our proposed tag can face degradation of antenna efficiency caused by dielectric behavior of other regions in the human body, such as the human wrist with six layers, dry skin, fat, muscle, bone cortical, cancellous, and bone marrow and chest model with three layers (skin, fat, and muscle) at the frequency of 915 MHz [30].

**Figure 18:**
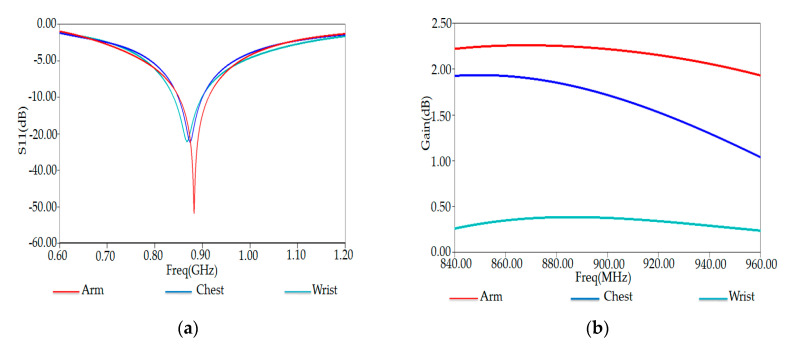
(**a**) Reflection coefficient S11 and (**b**) Gain versus frequency of proposed RFID tag placed on human wrist phantom.

Figure 18a,b show the simulated S11 and input impedance of our tag placed on the arm, wrist, and chest region of the human body in the simulated environment, respectively.

We remark a decrease of S11 in the case when the tag is placed on the chest and wrist part. Indeed, Electromagnetic (EM) properties of the human body depend on frequency and tissue types. According to their EM properties, human body tissues were categorized into high water content tissue (high dielectric constant and loss) and low water content tissue (low dielectric constant and loss). Fat, bone, and inflated lung are grouped with low water content tissue, while skin, muscle, and other main organs are grouped with high water content.

Moreover, we note that the gain reaches higher values for the arm because of the high water contents of fat tissue in the arm compared to the chest and the wrist. In our simulation, we have considered the dielectric behavior of the human wrist phantom. Table 5 lists the thicknesses of tissue layers (skin, fat, muscle, and bones) and the electromagnetic properties of human wrist phantom at a frequency of 915 MHz.

Figure 15a shows the simulated reflection coefficient S11 of our RFID tag attached to the human wrist phantom’s planar model. The reflection coefficient S11 reaches a value of −31.86 dB at resonance frequency 870 MHz.

In the following, we investigated an optimized tag antenna’s performance using both HFSS and CST simulation tools. The reflection coefficient S11 and total-gain against the frequency are respectively shown in Figure 18a,b. Here, we also notice that both solvers give similar results. The reflection coefficient S11 has a maximum of S11 = −32.85 dB at frequency resonance of 878 MHz. The extracted maximum gain from Figure 18b has a value of 1.82 dB around 878 MHz.

One can notice that the gain in arm and chest phantoms is much higher than that obtained in the wrist phantom. This can be related to the high water content in fat tissues in the arm and chest compared to the wrist. The gains obtained in the three cases (arm, wrist, and chest) are still higher, improving our wearable tag’s good functionality near the human body model [31,32,33].

**Figure 19:**
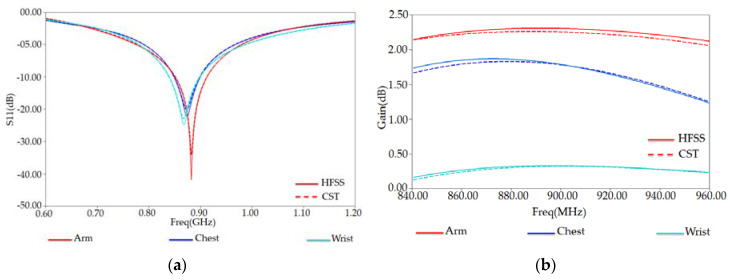
(**a**) Reflection coefficient S11 and (**b**) Gain versus frequency of proposed RFID tag placed on some human body regions (arm, chest, and wrist) in HFSS and CST solvers.

Figure 19 also shows the plots of S11 and gain obtained by the CST (blue curves) for comparison. One can remark that both HFSS and CST provide nearly similar results.

### Reading Range of RFID Tag in Arm, Chest, and Wrist

The maximum distance $d_{max}$ from which a tag can be activated by the RFID reader is proportionally dependent on the antenna radiation performances and can be derived from Friis free space equation [2]:

(12) $$d_{max}\left(\theta ,\phi \right)=\frac{C}{4\pi }\sqrt{\frac{EIRP_{R}}{P_{chip}}\tau G{\left(\theta ,\phi \right)}_{T}}$$

where $EIRP_{R}=G_{R}.P_{in}$. $P_{in}$ is the equivalent isotropic radiated power emitted by the reader and fixed according to the regulations of different countries. In particular, within the 865.6–867.6 MHz Europe RFID band, the $EIRP_{R}$ is fixed at 3.2 W, and within 902–928 MHz USA RFID band, the $EIRP_{R}$ is fixed at 4 W. $G{\left(\theta ,\phi \right)}_{\tau }$ is the realized gain $G{\left(\theta ,\phi \right)}_{\tau }=\tau \;.\;G{\left(\theta ,\phi \right)}_{T}$ given by the radiation gain of the tag antenna $G{\left(\theta ,\phi \right)}_{T}$ reduced by the power transfer coefficient *τ* between the tag antenna and the microchip.

**Figure 20:**
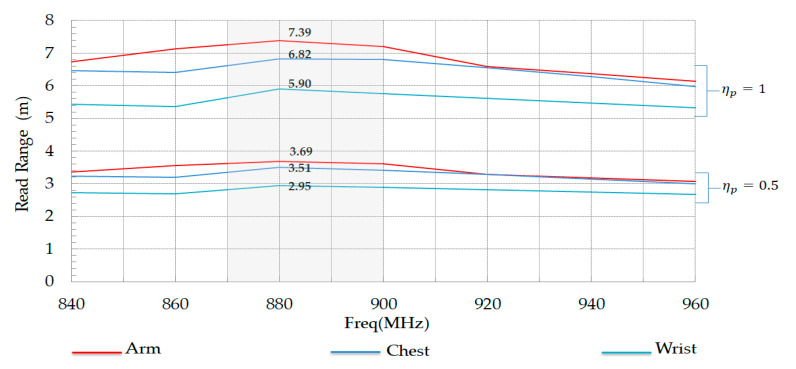
Read range of proposed tag on arm, wrist, and chest in the case of $\eta_{p}=0.5$ and $\eta_{p}=1$.

Figure 20 shows the simulated data of the theoretical read range between tag and reader. The best read range value of the tag is reached when placed on the planar form of the human arm phantom. The maximum read distance is almost 3.69 m in the case of a reader with circular polarization ($\eta_{p}=0.5$), and it becomes 7.39 m in the case of a reader with linear polarization ($\eta_{p}=1$).

## 5. Conclusions

We have presented our proposed antenna design, PSO optimization method, and robustness analysis of a five-slotted patch sensor tag with a flexible and biocompatible substrate. From our numerical results presented above, we have proved that the correct selection of antenna radiating part and ground plane dimensions lead to the best antenna radiation performances and good matching features. Indeed, our proposed tag’s calculated read range can be up to 4 m nearby of different parts of the human body. Our conceived wearable sensor tag operates in a universal UHF band for RFID biomedical sensing applications. It can host multiple sensors and other electronic components that make it a good candidate for tracking and monitoring patients’ biological parameters in healthcare environments.

## Figures and Tables

**Table 1:** Geometrical dimensions of proposed conventional RFID tag.

Dimension	Value(mm)
$W_{p},W_{s}$	90
$L_{p},L_{s}$	30
a	20
b	10
c	8
d	2
h	1

**Table 2:** Electrical properties of human arm phantom at 915 MHz.

Tissues	Dielectric Constant	Conductivity (S/m)	Loss Tangent
Skin	41.3	0.87	0.415
Fat	5.46	0.05	0.185
Muscle	55	0.94	0.339

**Table 3:** Range of variation of proposed antenna parameters.

Antenna Parameters	Lower Bound (mm)	Upper Bound (mm)	Step Size (mm)
$L_{p}$,${\;L}_{s}$	26	50	10
$W_{p}$, $W_{S}$	50	128	6
a	4	10	1
b	18	43	1
c	4	12	2
d	1	3	0.5
h	0.5	4	0.5

**Table 4:** Comparison between conventional antenna to PSO optimized antenna.

Dimension	Conventional Antenna Value (mm)	PSO-Optimized Antenna Value (mm)
$W_{p},W_{s}$	90	86
$L_{p},L_{s}$	30	26
a	20	19
b	10	8.45
c	8	9
d	2	2
h	1	2

**Table 5:** Comparison between conventional five-slot patch antenna and PSO-optimized antenna performances.

Parameter	Conventional Antenna	PSO-Optimized Antenna
S11 (dB)	−12.49	−50 dB
BW (%)	12.48	23.80
Gain (dB)	0.92	2.27

**Table 6:** Dimensions of the four-layer cylindrical model of the human arm.

Tissue Layer	Skin	Fat	Muscle	Bone
Arm model	4 mm	4 mm	23 mm	24 mm
